# Standard-length stems require lower distal filling ratios than short stems to achieve neutral alignment in reverse total shoulder arthroplasty

**DOI:** 10.1016/j.jseint.2026.101765

**Published:** 2026-07-24

**Authors:** David Hollo, Istvan Soproni, Felix Toft, Atesch Ateschrang, Igor Shirinskiy, William G. Blakeney, Stefan Bauer

**Affiliations:** a Department of Orthopedic Surgery and Traumatology Cantonal Hospital Aarau, Aarau, Switzerland; b Dr Manninger Jenö, Trauma Centre Budapest, Budapest, Hungary; c Centre de l'épaule, du coude et du Membre Supérieur, EHC, Morges, Switzerland; d Department of Human Movement Sciences, Vrije Universiteit Amsterdam, Amsterdam, The Netherlands; e Division of Surgery, UWA Medical School, University of Western Australia, Perth, Australia; f Department of Orthopaedics, Royal Perth Hospital, Australia School of Surgery, University of Western Australia, Perth, Australia; g Universitätspital Basel (USB), Surgical Outcome Research Centre (SORC), Basel, Switzerland

**Keywords:** Shoulder, Humeral stem, Reverse total shoulder arthroplasty, Coronal alignment, Distal filling ratio, Short stem, Malalignment

## Abstract

**Background:**

Short humeral stems in reverse total shoulder arthroplasty have been introduced to reduce stress shielding by decreasing distal filling ratios (DFRs). However, reduced diaphyseal engagement may compromise coronal alignment. This study compared coronal alignment between short and standard-length stems and identified implant-related and bone-related factors associated with malalignment.

**Methods:**

Consecutive patients undergoing primary reverse total shoulder arthroplasty with short or standard-length (long) Perform humeral stems (Stryker) were included from 2 institutional registries from 2023-2025. Standardized radiographs were analyzed retrospectively. Coronal alignment was measured on true anteroposterior radiographs at a minimum 6-month follow-up. Malalignment was defined as > 5° deviation. Pre-operative computed tomography assessed metaphyseal cancellous bone density and cortical thickness (Tingart method). Post-operative radiographs were used to measure DFR and metaphyseal collar filling ratio. Between-group comparisons were performed using the Wilcoxon rank-sum test. Multivariable linear regression evaluated the independent effect of stem type on alignment, adjusting for age, sex, metaphyseal cancellous bone density, and metaphyseal collar filling ratio. A sensitivity model incorporating DFR and stem length assessed the contribution of endosteal contact.

**Results:**

A total of 114 patients were included (57 short stems and 57 standard-length stems). Short stems demonstrated greater varus-valgus deviation than standard-length stems (median 3.50° [interquartile range: 1.90°-5.10°] vs. 1.90° [interquartile range: 1.00°-3.90°]; *P* = .002). Malalignment (> 5°) occurred in 26.3% of short stems and 12.3% of standard-length stems. In multivariable analysis, short stem use remained independently associated with increased deviation (β = 1.26, 95% confidence interval [CI]: 0.47-2.05; *P* = .002). In the sensitivity model, DFR was the strongest predictor of alignment (β = −7.99, 95% CI: −11.37 to 4.61; *P* < .001), while stem type remained significant (β = 1.65, 95% CI: 0.84-2.46; *P* < .001). Standard-length stems maintained stable alignment at lower DFRs, corresponding to an absolute risk reduction of 30.4% for malalignment (95% CI: 7.4%-50.9%; *P* = .012). An observed DFR of 48% was associated with consistent alignment for standard-length stems vs. 59% for short stems.

**Conclusion:**

This study demonstrates that standard-length humeral stems are associated with more consistent coronal alignment than short stems, although the clinical significance of this radiographic difference remains unknown. Standard-length stems demonstrated consistent alignment above an observed DFR of 48%, whereas short stems did so above 59%. Stem length independently influenced coronal alignment and allowed stable positioning despite lower DFRs. These findings suggest that greater stem length may permit reduced distal cortical engagement without compromising coronal alignment.

Reverse total shoulder arthroplasty (rTSA) has become an established treatment for a broad range of shoulder pathologies, including cuff tear arthropathy (CTA), massive irreparable rotator cuff tears, and complex degenerative conditions of the glenohumeral joint.^6^^,^^12^ Advances in implant design and surgical technique have resulted in improved clinical outcomes and implant survivorship. However, complications related to component positioning and fixation remain an important concern.^13^^,^^31^^,^^32^^,^^35^

Proper humeral component alignment in rTSA is critical for restoring shoulder biomechanics. Accurate positioning optimizes deltoid efficiency and minimizes complications such as instability, polyethylene wear, and periprosthetic fracture.^3^^,^^20^ Deviations in the coronal plane may have substantial consequences. Excessive varus or valgus positioning may adversely affect load transfer to the humeral cortex and metaphysis, compromising implant stability.^1^^,^^3^^,^^23^ Prior studies have demonstrated that humeral stem alignment influences the effective neck-shaft angle (NSA).^2^^,^^8^^,^^29^ This may influence deltoid vector alignment for balanced concavity compression and liner stability.^26^ Consequently, alignment may affect both radiographic and clinical outcomes.

The use of short stem humeral components in rTSA has increased in recent years. This shift is driven by the potential advantages of bone preservation, the facilitation of future revision surgery, and reduced stress shielding.^29^ Nevertheless, concerns persist regarding the ability of shorter stems to achieve reliable coronal plane alignment. This is particularly relevant in patients with compromised bone quality or limited distal fixation.^29^ In contrast, traditional longer stems may provide improved axial and coronal stability through increased diaphyseal engagement and press-fit. However, this stability comes at the cost of greater bone sacrifice associated with revision difficulties and potential stress shielding.^19^^,^^29^

In addition to stem length, there are specific anatomical factors that may influence stem alignment. Implant–bone interaction parameters such as distal filling ratio (DFR) and cortical thickness may also influence humeral component positioning following rTSA.^33^ Reduced metaphyseal or diaphyseal support may predispose stems to varus or valgus alignment. Such deviations can alter soft-tissue tensioning and joint stability. It is therefore important to understand how the loss of diaphyseal guidance in short stem designs affects the reproducibility of alignment compared to traditional stem length.

The purpose of this study was to evaluate post-operative humeral component alignment following rTSA using short and standard-length humeral implants. This study also aimed to identify implant-related and bone-related factors associated with coronal plane malalignment. We hypothesized that short stem implants would demonstrate greater varus-valgus angulation compared with standard-length stems and that reduced DFR and Tingart canal diameter would be associated with increased angular deviation.

## Materials and methods

### Study design and patient selection

This was a consecutive cohort study with retrospective analysis conducted in 2 centers that evaluated post-operative humeral component alignment following rTSA. Shoulder arthroplasty procedures performed between April 13, 2023 and June 30, 2025 at Kantonsspital Aarau in Aarau, Switzerland and Ensemble Hospitalier de la Côte in Morges, Switzerland were considered for inclusion. Patients were included if they underwent elective primary rTSA with the Stryker Perform humeral stem and had post-operative true anteroposterior (AP) radiographs with a minimum follow-up of 6 months. Exclusion criteria were revision arthroplasty and proximal humerus fractures. Study approval was obtained from respective cantonal Ethics Committees (Approval number: CER-VD 2023-01051; EKNZ-202502079), and all patients provided informed consent for retrospective analysis of their radiographs and data.

### Surgical technique and implants

All surgeries were performed by one of 3 fellowship-trained shoulder surgeons (S.B., F.T., D.H.) using a standard deltopectoral approach. Humeral components were implanted using an intramedullary alignment technique to perform the humeral osteotomies in all cases. Extra-long trial compactors were used for all size 2, 2+, 3, and 3+ stems for alignment guidance. Intraoperative fluoroscopic guidance was not used.

All rTSA were carried out using the Perform humeral stem (Stryker, Kalamazoo, MI, USA). The short stems are available in sizes 1 through 4 (as well as corresponding plus [+] sizes), which correspond to collar diameters of 32 mm, 34 mm, 38 mm, and 42 mm. They have overall lengths ranging from 56 mm to 77 mm and distal tip diameters ranging from 5 mm to 12 mm. The short stem uses a metaphyseal-based geometry featuring a medial collar for optimal load transfer to the native bone and a tapered distal stem designed to reduce the canal filling ratio. The proximal portion is coated with porous titanium to achieve biological fixation, while rotational stability is enhanced by lateral flat surfaces and a medial fin. The standard-length (long) stems are exclusively available in plus sizes (1+, 2+, 3+, and 4+), with lengths ranging from 89 mm to 116 mm and distal tip diameters ranging from 6 mm to 12 mm. These longer stems share the identical proximal metaphyseal geometry as the short stems, including the collar diameters, porous titanium coating, and rotational fins, but feature an extended diaphyseal segment designed to increase diaphyseal engagement. For both standard and short stem implantations (sizes 2, 2+, 3, and 3+), an extra-long standard compactor was used during the metaphyseal preparation step to maximize diaphyseal engagement and optimize coronal alignment.^16^

Stem selection was driven by institutional protocols rather than individual patient characteristics. At one participating center, an initial period of short stem usage was followed by a consecutive series of standard-length stem implantations after an institutional change in implant strategy. The second participating center used predominantly short stem implants throughout the study period. Implant configuration, including liner type (standard or retentive), liner angulation (0° or 10°), and glenosphere eccentricity, was selected during pre-operative planning and intraoperatively according to scapular morphology and surgeon judgment. The bony-increased offset reverse shoulder arthroplasty (BIO-RSA) technique and cemented fixation were used when indicated.

### Radiographic evaluation and data collection

Patient demographics (age at surgery, sex, and body mass index), pre-operative diagnosis, surgical center, and detailed implant characteristics (stem length category, numeric stem length, numeric distal tip diameter, cemented fixation, BIO-RSA technique, liner constraint, and glenoid baseplate type) were recorded from prospective institutional implant registries. The liner stability ratio (LSR) was calculated for all patients based on glenosphere size and jump height values previously reported for this implant system.^3^^,^^28^ All patients underwent standardized pre-operative and post-operative radiographic evaluation according to institutional protocols.

Pre-operative computed tomography (CT) scans, used for rTSA planning, were analyzed using Blueprint (Blueprint 3.2; Blueprint Software, Austin, USA) and Glenosys software (Glenosys 10.6.4; Imascap, Brest, France). Pre-operative CT was used to measure metaphyseal cancellous bone density (MCBD) (in Hounsfield units), anatomical head cut diameter, metaphyseal collar filling ratio (MCFR), and estimated Tingart canal diameter.

**Figure 1:**
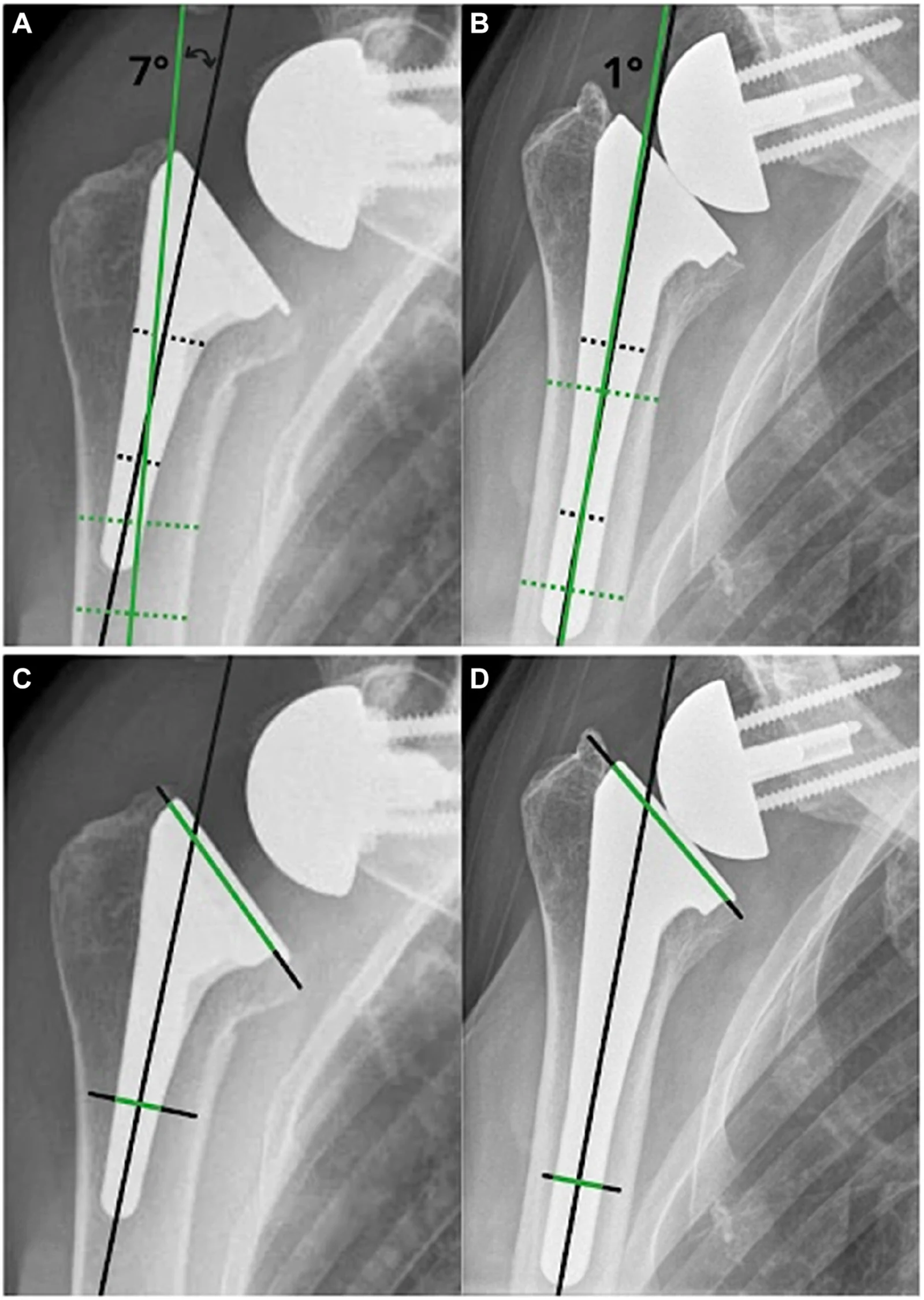
Measurement of humeral stem coronal alignment and filling ratios. (**A** and **B**) The angle between the longitudinal intramedullary humeral shaft axis (black line) and the central axis of the short and standard-length humeral implant stem (*green line*) defines the coronal alignment deviation. A counterclockwise rotation of the implant axis relative to the shaft axis in this right shoulder indicates valgus angulation. (**C** and **D**) Filling ratio measurements performed perpendicular to the intramedullary humeral shaft axis (*black line*). The metaphyseal collar filling ratio (*upper green and black line*) is measured at the level of the distal medial border of the stem. The distal filling ratio (*lower green and black line*) is measured 2.5 cm proximal to the distal tip of the stem. At each level, the filling ratio is calculated as the implant stem width divided by the endosteal canal width.

The primary outcome was post-operative humeral stem angulation, measured on the latest available standardized radiograph (minimum 6 months post-operatively) demonstrating a true AP position of the stem, defined as the proximal stem being displayed as a straight line as shown in Figure 1. Following the measurement principles described by Abdic et al, angular deviation and the DFR were calculated.^1^ The longitudinal intramedullary humeral shaft axis and the central axis of the humeral implant stem were identified. Coronal alignment was defined as the exact angle measured between these 2 axes. This parameter was categorized as neutral for angular deviations of 5° or less, and malalignment for varus or valgus deviations greater than 5°, consistent with previously published work evaluating humeral stem alignment in rTSA.^17^ Coronal malalignment exceeding 5° has been identified as the strongest independent predictor of proximal humeral stress shielding (odds ratio [OR]: 14.82) in reverse shoulder arthroplasty.^18^ The DFR was calculated to quantify diaphyseal engagement. A measurement line was established orthogonal to the intramedullary humeral shaft axis at a level 2.5 cm proximal to the distal tip of the prosthesis. At this specific level, the horizontal width of the implant stem and the inner endosteal width of the humeral cortex (from the medial to the lateral endosteal border) were measured. The DFR was then calculated as the quotient of the stem width divided by the endosteal intramedullary canal width. In cemented cases, the DFR was not recorded as cortical engagement is not required for fixation.

To ensure reliability, 2 independent observers performed all radiographic measurements in a blinded fashion (I.S. and D.H.) For both angular deviation and DFR measurements, if the interobserver difference was > 2° or > 2%, respectively, the radiograph was reassessed by a third author (S.B.).

### Statistical analysis

The distribution of continuous variables was assessed for normality using the Shapiro-Wilk test within each study group. Normally distributed variables were presented as mean and standard deviation (SD). Non-normally distributed variables were presented as median with interquartile range (IQR). Categorical variables were presented as counts with percentages. Interobserver reliability for the primary continuous radiographic measurements was assessed using the intraclass correlation coefficient (ICC), specifically using a 2-way mixed-effects model for absolute agreement. An ICC value greater than 0.80 was considered to indicate excellent agreement.

The primary unadjusted analysis evaluated differences in post-operative coronal plane alignment deviation between stem groups. Between-group differences were evaluated using the Student’s *t*-test for normally distributed continuous data, the Wilcoxon rank-sum test for non-normally distributed continuous data, and the Chi-square test for categorical data.

Univariable associations between baseline covariates and coronal alignment deviation were assessed using simple linear regression for continuous predictors and the Hodges-Lehmann estimator from the Wilcoxon rank-sum test for binary predictors. Numeric stem length was excluded a priori due to collinearity with stem group by design. A screening threshold of *P* value less than .10 was applied, with stem length group forced into all models regardless of *P* value. Two multivariable linear regression models were constructed: a primary model including stem length group, age, sex, MCBD, and metaphyseal collar filling index and a sensitivity model including stem length group and all variables meeting the screening threshold. Unstandardized regression coefficients with 95% confidence intervals (CIs), *P* values, and adjusted R-squared were reported. Residual normality was assessed using the Shapiro-Wilk test, and bias-corrected accelerated bootstrap CIs based on 10,000 replicates were computed when residuals were non-normal. To confirm robustness to the skewed, zero-bounded distribution of the outcome, 3 sensitivity analyses were performed: median quantile regression, robust regression using Huber M-estimation, and logistic regression using dichotomized malalignment (varus or valgus deviation greater than 5°) as the outcome.

An exploratory threshold analysis was performed to identify the observed DFR above which the malalignment rate was zero for each stem design. A Fisher’s exact test was used to compare outlier rates within the observed DFR range, from which the absolute risk reduction with 95% CI was calculated. These values were derived from the observed data and were not subjected to receiver operating characteristic analysis or validated in an independent cohort and are therefore presented as exploratory observations.

To assess the influence of non-randomized allocation, 3 sensitivity analyses were performed: the date of surgery (days since the first case) and surgical center were each added to the primary multivariable model, and a linear mixed-effects model with center as a random intercept was constructed to account for between-center clustering.

No a priori sample size calculation was performed, as all consecutive eligible patients within the study period were included. A post-hoc power analysis was conducted using the observed effect size for the primary outcome.

A *P* value of less than .05 was considered statistically significant for all analyses. All analyses were performed using RStudio (version 2026.01.1; Posit Software, PBC, Boston, MA, USA) with the boot package for bootstrap CIs.

## Results

### Patient characteristics

A total of 114 patients were included in this study. The overall mean age was 74.0 years (SD: 8.2), and 72 patients (63.2%) were female (Table I). The median body mass index was 27.5 kg/m^2^ (IQR: 24.3-32.0). Pre-operative diagnoses included CTA in 51 patients (44.7%), osteoarthritis in 32 (28.1%), massive rotator cuff tear (MRCT) in 28 (24.6%), and post-traumatic arthropathy in 3 (2.6%).

**Table I:** Baseline characteristics and comparison between short stem and standard-length groups.

Characteristic	Total (N = 114)	Short stem (n = 57)	Standard stem (n = 57)	*P* value
Patient demographics				
Age at surgery, yrs	74.0 ± 8.2	74.1 ± 8.1	73.9 ± 8.4	.892
Female sex, n (%)	72 (63.2)	33 (57.9)	39 (68.4)	.244
BMI, kg/m^2^	27.5 (24.3-32.0)	27.7 (24.6-30.9)	27.3 (24.2-32.0)	.943
Diagnosis				.291
Cuff tear arthropathy, n (%)	51 (44.7)	28 (49.1)	23 (40.4)	
Osteoarthritis, n (%)	32 (28.1)	14 (24.6)	18 (31.6)	
Massive rotator cuff tear, n (%)	28 (24.6)	15 (26.3)	13 (22.8)	
Post-traumatic, n (%)	3 (2.6)	0 (0.0)	3 (5.3)	
Implant characteristics				
Cemented fixation, n (%)	16 (14.0)	6 (10.5)	10 (17.5)	.419
BIO-RSA technique, n (%)	56 (49.1)	14 (24.6)	42 (73.7)	<.001
Liner angulation: angled 10°, n (%)	24 (21.2%)	22 (39.3%)	2 (3.5%)	<.001
Liner constraint: retentive (55%), n (%)	40 (35.1)	7 (12.3)	33 (57.9)	<.001
Liner stability ratio, n (%)	152 (147-194)	152 (147-152)	185 (152-205)	<.001
Glenoid baseplate: Perform, n (%)	58 (50.9)	43 (75.4)	15 (26.3)	<.001
Glenoid baseplate: Reversed II, n (%)	56 (49.1)	14 (24.6)	42 (73.7)	<.001
Radiographic measurements				
Coronal deviation: varus, n (%)	92 (80.7)	52 (91.2)	40 (70.2)	.009
Coronal deviation: valgus, n (%)	22 (19.3)	5 (8.8)	17 (29.8)	.009
Coronal deviation, °	2.7 (1.5-4.6)	3.5 (1.9-5.1)	1.9 (1.0-3.9)	.002
MCBD, HU	20.0 (7.8-38.2)	22.0 (10.0-40.5)	18.0 (7.0-29.0)	.328
AHCD, mm	46.3 (43.0-49.9)	46.6 (44.0-49.6)	46.0 (42.8-50.6)	.783
MCFR	0.76 ± 0.07	0.77 ± 0.07	0.75 ± 0.06	.129
Tingart canal diameter, mm	13.0 (12.0-14.0)	13.0 (12.3-13.9)	12.9 (12.0-14.2)	.783
Distal filling ratio	0.52 ± 0.11	0.53 ± 0.09	0.50 ± 0.11	.133

*BMI*, body mass index; *BIO-RSA*, bone-increased offset reverse shoulder arthroplasty; *MCBD*, metaphyseal cancellous bone density; *HU*, Hounsfield units; *AHCD*, anatomical head cut diameter; *MCFR*, metaphyseal collar filling ratio.

### Surgical and implant characteristics

A short stem humeral component was used in 57 patients (50.0%), and a standard-length stem was used in 57 patients (50.0%) (Table II). The mean numeric stem length for the entire cohort was 82.9 mm (SD: 17.4), with a mean numeric distal tip diameter of 8.4 mm (SD: 1.7). Humeral stems were cemented in 16 cases (14.0%). The BIO-RSA technique was used in 56 patients (49.1%). Glenoid baseplates consisted of the Perform design in 58 patients (50.9%) and the Reversed II design in 56 patients (49.1%). The liner insert was symmetric (0°) in 89 patients (78.8%) and angled (10°) in 24 patients (21.2%). Angled liners were used significantly more frequently in the short stem group than in the standard-length stem group (39.3% vs. 3.5%, *P* < .001). All 24 angled liner cases were implanted with a standard non-retentive insert, corresponding to an LSR of 144%-152%. Liner constraint was standard in 73 cases (64.0%) and retentive in 40 cases (35.1%); liner constraint data were unavailable for one patient. Among patients with a standard liner (n = 73), the median LSR was 152% (IQR: 147%-152%). Among patients with a retentive liner (n = 40), the median LSR was 205% (IQR: 194%-205%). The disproportionate use of angled liners in short stems reflected a center-level practice difference rather than stem-specific selection. Angled liners were used in 88.9% of cases at the second center vs. 1.1% at the primary center, and within each center, liner angulation was independent of stem length. Glenosphere eccentricity did not differ between stem groups (median 2.0 vs. 2.0 mm, *P* = .147).

**Table II:** Stem size distribution.

Characteristic	Total (N = 114)
Stem length and size, n (%)	
L1+	12 (20.5)
L2+	28 (24.6)
L3+	15 (13.2)
L4+	2 (1.8)
S1	4 (3.5)
S1+	3 (2.6)
S2	7 (6.1)
S2+	16 (14.0)
S3	6 (5.3)
S3+	13 (11.4)
S4	7 (6.1)
S4+	1 (0.9)

*L*, long stem; *S*, short stem.

### Pre-operative and post-operative measurements

Pre-operative CT analysis demonstrated a median MCBD of 20.0 HU (IQR: 7.8-38.2) and a median anatomical head cut diameter of 46.3 mm (IQR: 43.0-49.9). The mean MCFR was 0.76 ± 0.07. The median estimated Tingart canal diameter was 13.0 mm (IQR: 12.0-14.0). Post-operative radiographic evaluation revealed a mean DFR of 0.52 ± 0.11.

### Unadjusted coronal alignment comparison

**Figure 2:**
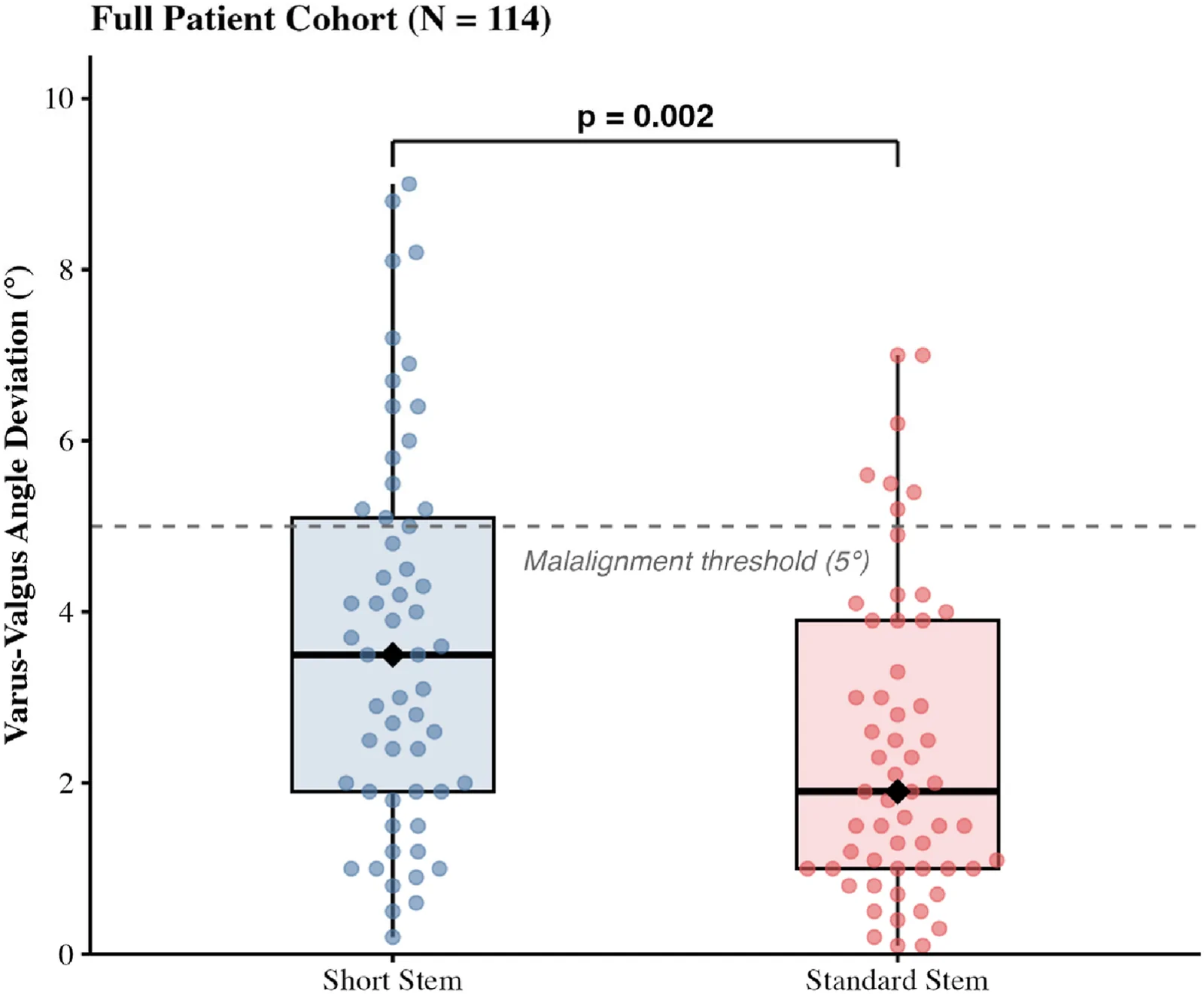
Comparison of coronal malalignment by stem type.

Coronal malalignment exceeding 5° was found in 15 shoulders of the short stem group (26.3%, 95% CI: 16.6%-39.0%) and in 7 patients in the standard-length stem group (12.3%, 95% CI: 6.1%-23.2%), corresponding to an OR of 2.55 (95% CI: 0.95%-6.84). The Hodges-Lehmann median difference in coronal alignment between groups was 1.2° (95% CI: 0.4°-2.0°), with a medium standardized effect size (Cohen's d = 0.62). An initial unadjusted comparison of coronal plane alignment was performed on the entire cohort (N = 114). The Shapiro-Wilk test confirmed non-normal distribution of varus-valgus angle deviation in both the short stem group (W = 0.955; *P* = .032) and the standard-length stem group (W = 0.906; *P* < .001); therefore, a non-parametric approach was adopted (Fig. 2). Patients receiving a short stem humeral component demonstrated a significantly greater median varus-valgus angle deviation compared to those receiving a standard-length stem (3.50° [IQR: 1.90°-5.10°] vs. 1.90° [IQR: 1.00°-3.90°]; W = 1,072; *P* = .002), with excellent interobserver reliability (ICC = 0.91, 0.88, and 0.85 for alignment, DFR, and MCFR, respectively). Varus deviation was more frequent in the short stem group (52/57, 91.2% vs. 40/57, 70.2%; *P* = .009), and among malaligned short stems, varus predominated (13/15, 86.7%). Coronal alignment did not differ significantly between cemented and cementless stems (median varus-valgus angle 3.4° [IQR: 2.0°-5.4°] vs. 2.5° [IQR: 1.1°-4.2°]; *P* = .215).

### Univariable screening

To identify candidate predictors for the multivariable model, univariable associations between each baseline covariate and post-operative coronal plane alignment deviation were assessed (Table III). For continuous predictors, simple linear regression was used. For binary predictors, the Hodges-Lehmann estimator derived from the Wilcoxon rank-sum test was reported as the location shift estimate given the non-normal distribution of the outcome. Three variables met the univariable screening threshold of *P* < .10: stem length group (short vs. standard; Hodges-Lehmann estimate = 1.20, 95% CI: 0.40-2.00, *P* = .002), liner constraint (retentive vs. standard; Hodges-Lehmann estimate = −0.60, 95% CI: −1.40 to 0.10, *P* = .089), and DFR (β = −6.93, 95% CI: −10.48 to 3.37, *P* < .001).

**Table III:** Univariable analysis.

Predictor	Method	Estimate	95% CI	*P* value
Age (per yr)	SLR	−0.008	−0.057 to 0.040	.739
Sex (female vs. male)	HL	−0.458	−1.200 to 0.300	.242
BMI (per kg/m^2^)	SLR	−0.017	−0.094 to 0.061	.667
Stem length (short vs. standard)	**HL**	**1.200**	**0.400 to 2.000**	**.002**
Cemented fixation (yes vs. no)	HL	0.800	−0.400 to 1.900	.215
BIO-RSA technique (yes vs. no)	HL	−0.200	−1.000 to 0.500	.477
Baseplate type (RII vs. Perform)	HL	−0.200	−1.000 to 0.500	.477
Liner constraint (retentive vs. standard)	**HL**	**−0.600**	**−1.400 to 0.100**	**.089**
Distal tip diameter (per mm)	SLR	−0.054	−0.294 to 0.186	.657
Metaphyseal cancellous bone density (per HU)	SLR	−0.002	−0.017 to 0.013	.746
Anatomical head cut diameter (per mm)	SLR	0.003	−0.045 to 0.051	.903
Metaphyseal collar filling ratio	SLR	2.248	−3.740 to 8.237	.458
Estimated Tingart canal diameter (per mm)	SLR	0.011	−0.162 to 0.183	.903
Distal filling ratio	**SLR**	**−6.925**	**−10.482 to -3.369**	**<.001**

*CI*, confidence interval; *SLR*, simple linear regression; *HL*, Hodges-Lehmann; *BMI*, body mass index; *BIO-RSA*, bony-increased offset reverse shoulder arthroplasty; *RII*, Reversed II; *HU*, Hounsfield units.

Association of candidate predictors with post-operative coronal plane alignment deviation. For continuous variables: unstandardized regression coefficient (β) from simple linear regression. For binary variables: difference in medians (Hodges-Lehmann estimator) from Wilcoxon rank-sum test, or mean difference from Welch *t*-test, depending on distributional assumption. Variables in bold entered the multivariable model (threshold *P* < .1). Treatment (stem length) was included as the primary exposure regardless of *P* value. Numeric stem length was excluded a priori from screening as it is collinear with stem group by design.

### Multivariable regression analysis

A multivariable linear regression model was constructed to evaluate the independent effect of stem type on varus-valgus angle deviation while adjusting for potential confounders. Stem length group (short vs. standard) was included as the primary exposure of interest. Age, sex, MCBD, and MCFR were included a priori on clinical grounds as potential confounders of the relationship between stem type and coronal alignment.

In the primary multivariable model, the use of a short stem humeral component was a statistically significant independent predictor of increased varus-valgus angle deviation (β = 1.26, 95% CI: 0.47-2.05, *P* = .002). Age (β = −0.001, 95% CI: −0.050 to 0.048, *P* = .957), sex (β = −0.47, 95% CI: −1.30 to 0.37, *P* = .269), MCBD (β = −0.006, 95% CI: −0.021 to 0.009, *P* = .437), and MCFR (β = 1.82, 95% CI: 4.44-8.08, *P* = .566) were not statistically significant predictors. The overall model explained a modest proportion of variance (adjusted R^2^ = 0.067, F (5,106) = 2.60, *P* = .029).

Adding the date of surgery to the primary model showed no temporal trend in alignment (β = −0.14 per year, 95% CI: −0.87 to 0.60, *P* = .707), and the addition of surgical center was likewise non-significant (β = −0.60, 95% CI: −1.69 to 0.50, *P* = .283). In a linear mixed-effects model with center modeled as a random intercept, short stem use remained significantly associated with increased varus-valgus angle deviation (β = 1.30, 95% CI: 0.30-2.31, *P* = .011), with an ICC for center of 0.007, indicating that less than 1% of the variance in coronal alignment was attributable to between-center differences.

A sensitivity model incorporating the univariable screening-derived predictors alongside stem group was constructed. In this model, the DFR emerged as the strongest predictor of coronal alignment deviation (β = −7.99, 95% CI: −11.37 to 4.61, *P* < .001), while stem group remained a significant independent predictor (β = 1.65, 95% CI: 0.84-2.46, *P* < .001). Liner constraint entered the model via univariable screening but did not reach statistical significance in the multivariable model (β = 0.35, 95% CI: 0.49-1.18, *P* = .414), suggesting its univariable association is accounted for by the other predictors. This model demonstrated superior explanatory capacity compared with the primary model (adjusted R^2^ = 0.222, F (3,108) = 11.57, *P* < .001).

Residual diagnostics revealed non-normal residuals for both the primary model (Shapiro-Wilk W = 0.952, *P* < .001) and the sensitivity model (Shapiro-Wilk W = 0.966, *P* = .006). To assess the robustness of inference under this assumption violation, bias-corrected and accelerated bootstrap CIs (10,000 replicates) were computed. For the primary model, the bootstrap 95% CI for the stem group coefficient was 0.50-2.04, confirming the significance and direction of the primary finding. For the sensitivity model, the bootstrap 95% CIs were: stem group 0.80-2.58, DFR −11.56 to 4.23, and liner constraint 0.49-1.30. All bootstrap intervals were consistent with the parametric estimates, confirming that the non-normal residuals did not materially affect the inference. The primary finding was robust to the distributional characteristics of the outcome. Short stem use remained significantly associated with increased coronal alignment deviation under median quantile regression (β = 1.63, 95% CI: 0.62-2.63, *P* = .002) and robust regression (β = 1.20, 95% CI: 0.41-1.99, *P* = .003). Logistic regression using dichotomized malalignment (> 5°) demonstrated a consistent direction of effect (adjusted OR: 2.57, 95% CI: 0.91-7.30, *P* = .076).

### Observed diaphyseal stability thresholds

**Figure 3:**
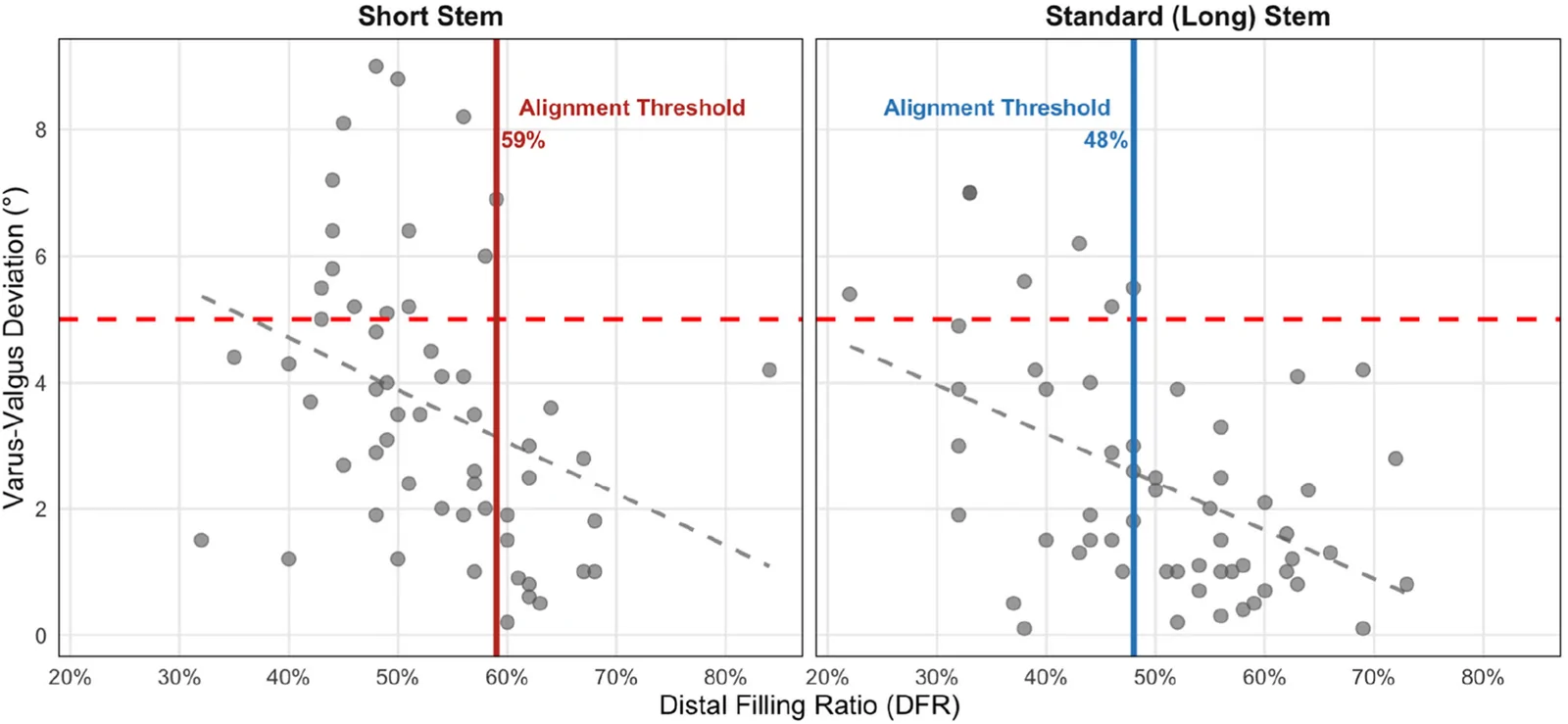
Threshold analysis of DFR and coronal plane alignment deviation for short and standard-length stems. The red dashed horizontal line indicates the 5° malalignment threshold. The solid vertical lines represent the stability tipping points where the malalignment rate is zero. *DFR*, distal filling ratio.

An observed stability threshold was identified for the standard-length stem at 48% DFR (Fig. 3). Every standard-length stem above this observed value maintained coronal alignment within 5°. Short stems demonstrated coronal alignment exceeding 5° up to a filling ratio of 59%. Within the filling range of 49%-59%, the standard stem demonstrated no case with coronal alignment exceeding 5° compared to a 30% rate of coronal alignment exceeding 5° for short stems corresponding to an absolute risk reduction of 30.4% (95% CI: 7.4%-50.9%; *P* = .012).

### Statistical power

For the primary outcome, the observed standardized effect size was medium (Cohen's d = 0.62), corresponding to a post-hoc statistical power of 90.5% with 57 patients per group at an alpha of 0.05.

### Complications

Overall, 7 patients (6.1%) experienced complications requiring clinical management or revision surgery. One patient (0.9%) sustained an acromial spine fracture managed non-operatively with a satisfactory clinical outcome and no surgical revision.

Six revision procedures were performed in 5 patients (4.4%) for post-operative instability. All 5 instability cases occurred in shoulders with a diagnosis of MRCT or CTA.

Four patients at the primary center developed post-operative instability, all with a 135° NSA and a standard 45% liner (LSR: 147%-152%). This corresponds to a dislocation rate of 11.4% (4/35) among CTA/MRCT shoulders treated with this implant configuration. After 35 shoulders with the diagnoses CTA/MRCT, the surgeon transitioned from a 45% to a 55% liner for cuff-deficient shoulders. Five revision procedures were required in these 4 patients, as one patient underwent 2 revisions including an additional stem revision due to 6° of varus stem alignment. The fifth instability case occurred at the second center in a shoulder with MRCT, a 135° NSA stem with a 10° angled liner (resulting in an effective implant inclination of 145° and LSR of 152%), and approximately 2° of varus angulation. Revision surgery in this patient was subsequently complicated by a periprosthetic joint infection necessitating 2 additional procedures with exchange of the mobile components. All 5 patients with instability were ultimately revised with a more retentive 55% liner of increased thickness to enhance concavity compression (LSR of 194%-205%), without changes to the glenosphere.

Two periprosthetic joint infections (1.8%) were observed. One occurred as a secondary complication following revision for instability as described above. The second was treated with a one-stage revision arthroplasty.

## Discussion

The findings of this study demonstrate that standard-length stems within this implant system are associated with a significantly lower propensity for coronal malalignment compared with short stems. To our knowledge, this is the first study evaluating the standard-length humeral component of this design (Perform nomenclature: long stem). The reduced risk of varus-valgus deviation suggests that longer stems achieve more consistent and predictable positioning, even at relatively low DFR. Specifically, our data indicate that an observed DFR as low as 49% was associated with maintained alignment for standard-length stems, whereas short stems in this cohort descriptively did so only above 60%. Even when using the recommended long standard compactor technique, short stems in this series showed a higher rate of coronal malalignment compared to standard-length stems (26.3% vs. 12.3% > 5°), suggesting that technique optimization alone may be insufficient to fully overcome the alignment challenges of metaphyseal-only fixation in short humeral stems.

Recent literature has highlighted that stem length is an important determinant of coronal alignment.^18^ The results of our study are consistent with this, and we hypothesize that the extended diaphyseal geometry of the standard-length stem may promote more consistent alignment by referencing the long axis of the humeral shaft. This is supported by radiographic analyses demonstrating that the humeral canal becomes more cylindrical and consistent in diameter at greater distances from the tuberosity, inherently favoring longer stems for alignment reliability.^5^ In contrast, shorter humeral components rely more heavily on metaphyseal fit and have a higher sensitivity to the initial starting point for intramedullary alignment of the osteotomy and manual broach orientation. Consequently, short stem implants may be more dependent on a higher DFR to maintain alignment. However, as demonstrated by Raiss et al, increasing the DFR to maximize endosteal contact can lead to higher rates of radiographic bone adaptations and stress shielding, including cortical osteopenia, spot welds, and condensation lines.^30^ Conversely, a low DFR carries its own risks: in a study of 44 shoulders implanted with the same humeral stem used in the current series, inferior migration of the stem exceeding 5 mm was observed in 13.6% of cases and was independently associated with both greater coronal malalignment and a lower proximal filling ratio, further underscoring the mechanical significance of adequate metaphyseal and diaphyseal engagement in short stem implants.^27^ Furthermore, coronal malalignment directly alters the effective NSA which may influence stress distribution in the proximal humerus, potentially increasing the risk of stress shielding^1^ and decreasing angular corrected LSR (Modelhart, Werthel, Athwal, and Moroder under review). This phenomenon is particularly concerning in osteoporotic bone, where altered load transfer can accelerate proximal bone resorption.^18^^,^^22^^,^^24^

Despite the higher radiographic incidence of coronal malalignment associated with short stems, current evidence does not consistently demonstrate inferior clinical outcomes. A systematic review of short stem rTSA comprising 555 shoulders reported significant improvements in range of motion and functional scores across all included studies, with no stem loosening recorded at any final follow-up and clinical outcomes comparable to standard-length designs at short-term follow-up.^34^ Clinical studies with follow-up periods of up to 5 years report stable fixation, low rates of aseptic stem loosening, and satisfactory functional outcomes, even in elderly patients and those with mild coronal deviations.^9^^,^^11^^,^^14^ However, the complication profile of short stems remains an area of active investigation. The incidence of periprosthetic fractures in short stem rTSA has been reported to be 3%.^21^ Recent comparative data suggest that periprosthetic fractures around short stems are more likely to result in prosthetic instability than those around standard-length stems, with revision arthroplasty more frequently required in the short stem group.^10^ Whether the altered metaphyseal stress distribution associated with coronal malalignment directly increases this fracture risk remains to be established, warranting further long-term investigation. Ongoing surveillance and comparative studies are necessary to establish their long-term survivorship and optimal indications.

The instability findings of this study carry important implications for implant selection and pre-operative planning. All 5 post-operative dislocations occurred exclusively in shoulders with a diagnosis of CTA or MRCT. This observation is consistent with a large multicenter study which identified a pre-operative diagnosis of rotator cuff arthropathy or MRCT as an independent predictor of dislocation, with an OR of 2.91, and reported that rTSA performed for osteoarthritis demonstrated lower dislocation rates than those for rotator cuff disease.^15^ Boubekri et al have recently published independent results from the United States with the same implant system reported in this study.^7^ Notably, the instability rate was higher than 10% in patients with cuff deficient shoulders and low liner constraint combined with a 135° NSA.^4^^,^^7^ This diagnosis-dependent instability risk could be explained by the principle of concavity compression as described by Lippitt and Matsen.^26^ In the absence of an intact rotator cuff, the compressive force directed into the glenosphere is reduced, making the LSR the deciding factor of joint stability.^5^ The lower NSA increases this risk as 135° NSA places higher demands on liner constraint than traditional 155° designs, and varus stem malalignment reduces the effective NSA further, with short stems in this series reaching effective NSA values as low as 126°.^3^ In this study, all 5 unstable rTSAs (LSR of 147%-152%) were successfully stabilized with more retentive liners (LSR of 194%-205%), which increase concavity depth to restore stability. Because more retentive 135° liners maintain equivalent impingement-free range of motion compared to standard 145° liners, they should be considered a primary and not a salvage option for shoulders with high instability risk.^2^ In cuff-deficient shoulders, there has been a change of implantation strategy to higher liner stability and capture ratios of 55% and the manufacturer has recently introduced an additional, more retentive liner option (65% capture ratio) for this 135° NSA implant system.

When selecting an implant, surgeons must weigh the risk of radiographic malalignment against the well-documented biomechanical consequences of longer stems. Standard-length stems offer distinct alignment reliability but are historically associated with higher rates of proximal stress shielding and calcar resorption associated with distal press-fit fixation, which may have negative implications for long-term bone health.^25^ Our study demonstrates that distal press-fit fixation can safely be avoided for longer standard-length stems with a minimal tolerable DFR to maintain alignment of 49%. Conversely, while malalignment in short stems can cause radiographic bone adaptations, these changes have not yet demonstrated to influence clinical outcomes in the short to medium term. Both stem types adequately restore humeral anatomy. Ultimately, the choice between these designs requires balancing the greater alignment consistency observed with standard stems against potential bone-length sparing benefits of the short stem, carefully tailored to the patient’s specific anatomy and bone quality. Gaps and future directions include the lack of long-term data for newer short and stemless designs.

### Limitations

This study has several limitations. First, the retrospective and non-randomized design introduces a potential selection bias regarding stem length. However, this risk was substantially mitigated by how the implants were used in clinical practice. Stem selection was primarily driven by evolving institutional protocols, which included a complete sequential transition to longer standard-length stems at one participating center. Because the choice of implant was based on these institutional changes rather than individual surgeon preference, the risk of allocation bias related to patient bone quality or specific anatomical challenges was minimized. Furthermore, this was a 2-center study including 3 surgeons, which could potentially limit the generalizability of the findings to the broader orthopedic community, particularly regarding variations in surgeon volume and the use of different implant systems. Consequently, while the findings may disproportionately reflect the outcomes of specialized, high-volume clinicians, this multicenter approach significantly reduces the bias inherent to single-surgeon series and enhances external validity. Additionally, the primary regression model explained only a modest proportion of the overall variance in coronal alignment, indicating that most of the variance was attributable to factors not captured in this analysis. Intraoperative surgical factors such as broaching trajectory, the direction and force of stem insertion, and use of fluoroscopy during insertion, together with unmeasured anatomical factors including humeral deformity, canal flare index, and residual osteophytes, represent unmeasured confounders that may independently influence coronal alignment and cannot be assessed from radiographic data alone. Another limitation is the reliance on 2-dimensional radiographic data. Coronal alignment was measured using standard AP plain radiographs. It is known that 2-dimensional projections are highly sensitive to humeral rotation, which can artificially project a false varus or valgus measurement. Although this risk was mitigated by analyzing true AP radiographs in which the proximal stem was displayed as a straight line, rotational malposition cannot be fully excluded, and alignment was not validated against 3-dimensional CT. The absence of CT-based validation therefore remains a limitation, and the reported coronal deviations should be interpreted as radiographic estimates. Furthermore, coronal alignment was assessed using the radiograph at a minimum of 6 months post-operatively rather than immediate post-operative imaging. While this reflects the clinically established alignment after early bone adaptation, it introduces the possibility that secondary positional changes, including stem subsidence previously reported in short stem rTSA, may have contributed to the observed differences between stem types. Moreover, this study is limited strictly to radiographic data. The clinical impact of coronal malalignment on patient-reported outcomes, range of motion, or pain was not assessed, and therefore, no direct causal link between the observed initial coronal deviations and long-term functional failure or aseptic loosening can be established from the current data. While the clinical significance of the observed malalignment remains a subject of active investigation, future prospective studies incorporating clinical outcome measures are warranted to determine whether the radiographic differences identified in this study translate into clinically meaningful differences in function or implant survival.

## Conclusion

This study demonstrates that standard-length humeral stems are associated with more consistent coronal alignment than short stems, although the clinical significance of this radiographic difference remains unknown. Standard-length stems demonstrated consistent alignment above an observed DFR of 48%, whereas short stems did so above 59%. Stem length independently influenced coronal alignment and allowed stable positioning despite lower DFRs. These findings suggest that greater stem length may permit reduced distal cortical engagement without compromising coronal alignment.

## Disclaimers:

Funding: No funding was disclosed by the authors.

Conflicts of interest: William G. Blakeney reports that he is a consultant for Enovis/Lima.

Stefan Bauer reports that he is a consultant for Stryker and Enovis.

Any additional authors, their immediate families, and any research foundations with which they are affiliated have not received any financial payments or other benefits from any commercial entity related to the subject of this article.

## Availability of data and materials

The datasets used and/or analyzed during the present study are available from the corresponding author on reasonable request.
